# Comparisons of care practices for very preterm infants and their short-term outcomes in two tertiary centers in northwest and south China: A retrospective cohort study

**DOI:** 10.1186/s12887-022-03623-5

**Published:** 2022-10-21

**Authors:** Haibo Peng, Yanling Shi, Fei Wang, Zhenchao Jin, Cungui Li, Jing Kang, Guofei Zhang, Lian Zhang, Yanli Yao, Zhangbin Yu

**Affiliations:** 1 Department of Neonatology, Shenzhen Baoan Women’s and Children’s Hospital, Jinan University, Shenzhen, China; 2 Department of Neonatology, Qinghai Red Cross Hospital, Xining, China; 3 Department of Neonatology, Shenzhen People’s Hospital, The Second Clinical Medical College of Jinan University, First Affiliated Hospital of Southern University of Science and Technology, Shenzhen, China; 4 Shenzhen, China

**Keywords:** Infant, Very preterm, Care practices, Obstetric, Delivery room, Mortality, Morbidity, China

## Abstract

**Background:**

Care practices for very preterm infants and the mortality and morbidity of the infants vary widely among countries and regions with different levels of economic development, including the different areas in China. We aimed to compare the obstetric and delivery room practices of two representative tertiary newborn centers in the northwestern and southern regions of China and the mortality and morbidity of their very preterm infants.

**Methods:**

A retrospective cohort study was conducted. Very preterm infants born between 22^0/7^ and 31^6/7^ weeks of gestation, and admitted to Qinghai Red Cross Hospital (QHH) and Shenzhen Baoan Women’s and Children’s Hospital (SZH) from January 1, 2018 to December 31, 2020, were included. The infants’ characteristics and short-term outcomes, and the hospitals’ care practices were compared between the two cohorts.

**Results:**

Three hundred and two infants in QHH and 505 infants in SZH were enrolled, and the QHH cohort was more mature than the SZH cohort was (gestational age 30.14 (29.14–31.14) vs. 29.86 (27.86–31.00 weeks, respectively), *p* < 0.001). Fewer antenatal steroids and more tracheal intubations were used in QHH than in SZH [(73.8% vs. 90.9%, *p* < 0.001) and (68.2% vs. 35.0%, *p* < 0.001, respectively)]. The odds of mortality [aOR = 10.31, 95%CI: (6.04, 17.61)], mortality or major morbidity [aOR = 5.95, 95%CI: (4.05, 8.74)], mortality despite active treatment [aOR = 3.14, 95%CI: (1.31, 7.53)], mortality or major morbidity despite active treatment [aOR = 3.35, 95%CI: (2.17, 5.17)], moderate or severe bronchopulmonary dysplasia [aOR = 3.66, 95%CI: (2.20, 6.06)], and severe retinopathy of prematurity [aOR = 3.24, 95%CI: (1.19, 8.83)] were higher in the QHH cohort. No significant difference in the rate of severe neurological injury or necrotizing enterocolitis ≥ Stage 2 was found between the cohorts.

**Conclusion:**

Obstetric and delivery room care practices used in the management of very preterm infants differed considerably between the QHH and SZH cohorts. Very preterm infants born in QHH have higher odds of mortality or severe morbidity compared with those born in SZH.

## Background

Very preterm infants (VPIs), those born earlier than 32 weeks of gestation, are at high risk of neonatal mortality, morbidity, and long-term neurodevelopmental disabilities [1]. Though care practices for VPIs and their mortality and morbidity have improved gradually in the last several decades, there is wide variation among countries and regions [2–4]. China is one of the largest developing countries in the world, with considerable economic imbalances among different regions. Studies from most of the more developed areas of China reveal improvements in caring for VPIs [2], whereas few studies from northwestern China are available.

Xining city, which is located on the Qinghai-Tibet Plateau, with an altitude of 2275 m above sea level, is representative of a city in northwestern China with lower economic development. Shenzhen city, which is in southern China, with an altitude of approximately 100 m above sea level, is representative of a city with a higher level of economic development. Thus, we hypothesized that there are variations in the care practices of newborn centers and the short-term outcomes of VPIs between these two cities. Therefore, we collected and analyzed clinical data from hospitals in these two cities of China, which are representative of two different levels of economic development, to compare the care practices of their newborn centers and the major outcomes of their VPIs.

The main objectives of this study were to determine the extent of differences, if any, in the obstetric and delivery room care practices of two newborn centers, examine the mortality and morbidity of their VPIs, and identify areas for improvement in the two newborn centers, with the goal of facilitating their learning from each other.

## Methods

### Study design

This retrospective cohort study was conducted at two hospitals: Qinghai Red Cross Hospital (QHH) and Shenzhen Baoan Women’s and Children’s Hospital (SZH). A protocol was registered in ClinicalTrials.gov (identifier NCT05116670). The study was approved by Qinghai Red Cross Hospital Ethics Committee and Shenzhen Baoan Women’s and Children’s Hospital Ethics Committee. As a public entity, QHH serves all socioeconomic strata of Qinghai Province, including a large number of minorities. Moreover, the majority of most critically ill pregnant women in Qinghai Province are transferred to QHH, which owns the largest tertiary newborn care center in Qinghai Province, with 48 intensive care beds. As a regional care center for critically ill pregnant women, SZH has a regional critical newborn care center with 38 intensive care beds. Between 2018 and 2020, approximately 8000 infants were delivered and 110 VPIs were admitted annually to QHH, and 19,000 infants were delivered and 200 VPIs were admitted annually to SZH.

### Study population

The study population consisted of VPIs born between 22^0/7^ and 31^6/7^ weeks of gestation (WG) and admitted to both centers over a 3-year period, from January 1, 2018 to December 31, 2020. Infants that were not born at QHH or SZH (Outborns), those who were admitted after 7 days of life, and those who had major congenital anomalies, were excluded from the study.

### Definitions

Gestational age (GA) was determined according to the first day of the last menstrual period, an early prenatal ultrasound examination, and a physical examination of the infant after birth. Maternal hypertension included maternal hypertension, chronic or pregnancy-induced, with or without edema and proteinuria. Maternal diabetes mellitus included maternal diabetes of any type or severity. Maternal chorioamnionitis was diagnosed clinically during pregnancy, labor, or delivery. Small for gestational age (SGA) was defined as a birth weight below the 10th percentile, based on the Fenton Growth Chart [5]. Preterm premature rupture of the membranes (PPROM) was defined as the rupture of the fetal membranes before 37 completed WG [6].

Care practices included antenatal steroid use, the use of antenatal magnesium sulfate, cesarean section, and delivery room resuscitation. Antenatal steroid use was defined as the administration of at least one dose of corticosteroid intravenously or intramuscularly to the mother at any time prior to delivery. Antenatal magnesium sulfate was defined as the intravenous administration of magnesium sulfate to the mother during pregnancy at any time before delivery.

The major outcomes were mortality, the composite outcome of mortality or major morbidity, including severe neurological injury (intraventricular hemorrhage (IVH) grade 3 or 4 based on Papile’s criteria [7], or any grade of periventricular leukomalacia determined by cranial imaging); necrotizing enterocolitis (NEC) (Stage 2 or higher based on Bell’s Staging Criteria [8]); moderate or severe bronchopulmonary dysplasia (BPD) (defined as requiring respiratory support with a nasal cannula > 2 L/min or noninvasive positive airway pressure or invasive mechanical ventilation at 36 weeks postmenstrual age (PMA) [9]); and severe retinopathy of prematurity (ROP) (Stage III or above [10] or the need for treatment). Early-onset sepsis was defined as confirmed sepsis on or before 72 h of life. Late-onset sepsis (LOS) was defined as confirmed sepsis acquired after 72 h of life. As a considerable proportion of very preterm infants were discharged against medical advice, we evaluated the mortality of the infants with active treatment, which meant that the infants received full treatment and were discharged in accordance with medical advice.

### Data collection and analysis

Data abstractors collected the data from both centers, which included information pertaining to maternal characteristics, neonatal characteristics, obstetric practices, delivery room practices, and neonatal short-term outcomes. The anonymized data were sent to the newborn center at SZH, and analyzed.

### Statistical analysis

Categorical variables were expressed as proportions n/N (%) and compared using the Chi-square (χ^2^) test. Continuous variables were expressed as (mean ± SD) or median and interquartile range (P25, P75), and compared using Student’s *t*-test or a nonparametric test, as appropriate. Two-tailed *p*-values < 0.05 were considered statistically significant. Patients were stratified by GA into four subgroups (22^0/7^–25^6/7^ WG, 26^0/7^–27^6/7^ WG, 28^0/7^–29^6/7^ WG, and 30^0/7^–31^6**/**7^ WG) for subgroup analyses. Logistic regression analysis was used to compare outcomes of very preterm infants at the two centers after controlling for relevant covariates that differed significantly between the two groups. Statistical analyses were performed using IBM SPSS Statistics, version 20.

## Results

### Patient characteristics

**Fig. 1:**
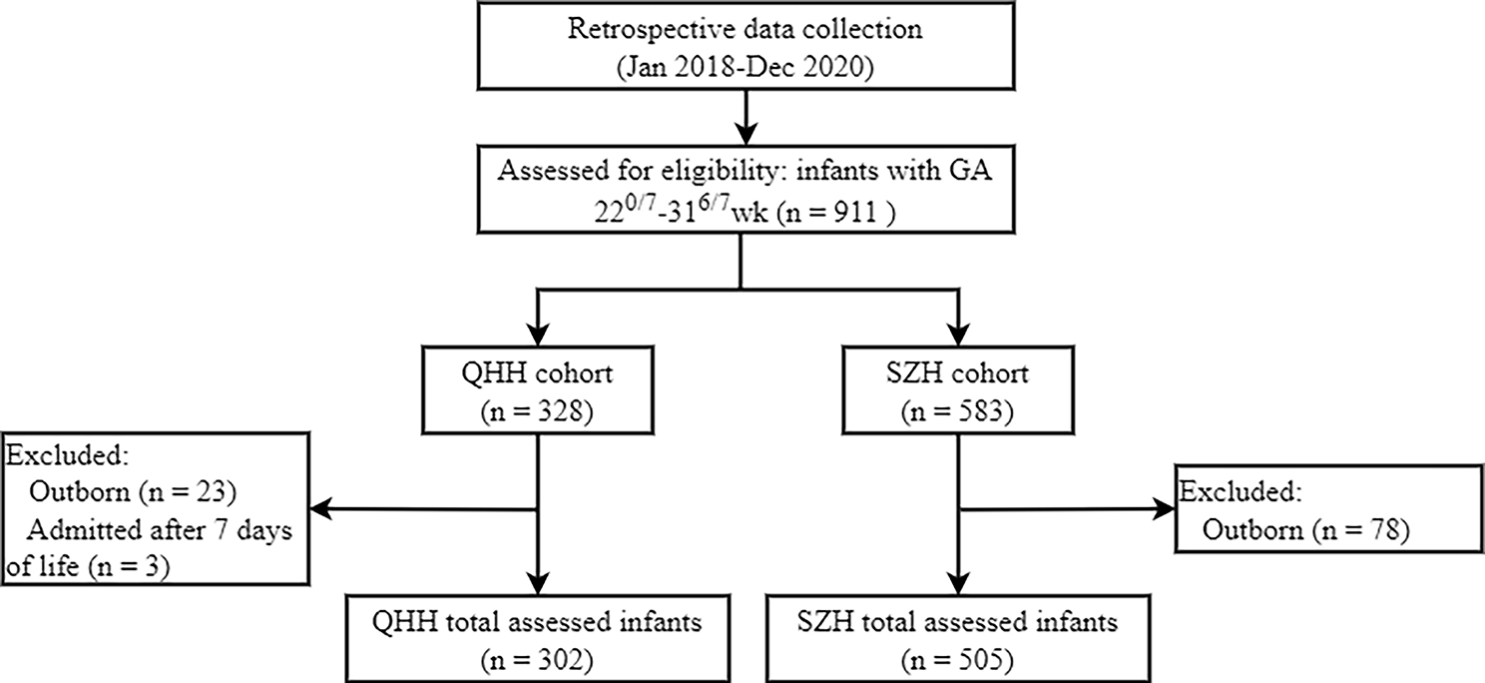
Flowchart of patient inclusion

A total of 807 preterm infants from 22^0/7^ to 31^6/7^ WG were included in the study, of which 302 were admitted to QHH, and 505 were admitted to SZH. A flowchart of the selection of patients is shown in Fig. 1.

Comparisons of maternal and neonatal characteristics between the two cohorts are presented in Table 1. The maternal age of the QHH cohort was significantly younger than that of the SZH cohort; but no significant differences in pregnancy complications (gestational hypertension, gestational diabetes mellitus, or clinical chorioamnionitis) between the cohorts were found. No difference in the rate of PPROM > 18 h was found between the two cohorts; 47.4% of the QHH mothers were ethnic minorities and 100% of the SZH mothers were Han. The rates of multiple pregnancies and use of assisted reproduction by the SZH mothers were significantly higher than the rates of the QHH mothers. The median GA of the infants in SZH was significantly lower than that of the infants in QHH: the minimum GA of the SZH infants was 22 WG, and the minimum GA of the QHH infants was 26 WG. The median birth weight of the SZH infants was significantly lower than that of the QHH infants, with a minimum birth weight of 400 g for the SZH cohort, and a minimum birth weight of 560 g for the QHH cohort. The distribution of infants in the different GA categories differed slightly between the two cohorts, especially the rates of infants younger than 28 WG. There was no difference in the gender proportions between the two cohorts, with males being 59.6% of the QHH cohort and 55.4% of the SZH cohort, and no significant difference in the 5-minute Apgar score was found between the two cohorts.

**Table 1:** Maternal and neonatal characteristics

Characteristic	QHH (N = 302)	SZH (N = 505)	***P*** value
Maternal age (years)	29(26, 32)	31(28, 34)	< 0.001
Maternal hypertension	53(17.5)	82(16.2)	0.629
Maternal diabetes	41(13.6)	80(15.8)	0.383
Chorioamnionitis	19(6.3)	45(8.9)	0.183
PPROM > 18 h	68(22.5)	142(28.1)	0.079
Multiple pregnancy	66(21.9)	150(29.7)	0.015
IVF	27(8.9)	89(17.6)	0.001
Ethnic Minority	143(47.4)	0(0)	< 0.001
Gestational age (weeks)	30.14(29.14, 31.14)	29.86(27.86, 31.00)	< 0.001
22^0/7^ − 25^6/7^	0(0)	38(7.5)	< 0.001
26^0/7^ − 27^6/7^	17(5.6)	94(18.6)	
28^0/7^ − 29^6/7^	105(34.8)	124(24.6)	
30^0/7^ − 31^6/7^	180(59.6)	249(49.3)	
Birth weight (g)	1330(1130, 1560)	1290(990, 1560)	0.004
< 500	0(0)	2(0.4)	< 0.001
500–749	5(1.7)	34(6.7)	
750–999	25(8.3)	97(19.2)	
1000–1249	83(27.5)	101(20.0)	
1250–1499	93(30.8)	121(24.0)	
1500–1999	88(29.1)	140(27.7)	
2000–2499	8(2.6)	10(2.0)	
Male gender	180(59.6)	280(55.4)	0.248
SGA	14(4.6)	14(2.8)	0.162
5-min Apgar score	8(6, 8)	8(7, 8)	0.476

QHH: Qinghai Red Cross Hospital; SZH: Shenzhen Bao’an Women’s and Children’s Hospital; PPROM: preterm premature rupture of the membranes; IVF: in vitro fertilization; SGA: small for gestational age

Data are presented as N (%) or median (P25, P75)

### Obstetric and delivery room practices

Comparisons of obstetric and delivery room practices between the two cohorts are presented in Table 2. The SZH used significantly more antenatal steroids for the 26^0/7^–31^6/7^ WG infants, significantly more magnesium sulfate for the 26^0/7^–27^6/7^ WG subgroup, and performed significantly more cesarean sections for the 28^0/7^–29^6/7^ WG subgroup. In the delivery room, QHH used significantly more facemask positive-pressure ventilation, and tracheal intubations for the 28^0/7^–29^6/7^ WG and 30^0/7^–31^6/7^ WG subgroups. A significantly larger number of infants in QHH were resuscitated using chest compressions and epinephrine in the delivery room.

**Table 2:** Obstetric and delivery room practices

Variable	Center	22^0/7^ − 25^6/7^ wk	26^0/7^ − 27^6/7^ wk	28^0/7^ − 29^6/7^ wk	30^0/7^ − 31^6/7^ wk	All VPIs
No. of infants	QHH	0	17	105	180	302
	SZH	38	94	124	249	505
Antenatal steroid	QHH	NA	7(41.2)	76(72.4)	140(77.8)	223(73.8)
	SZH	27(71.1)	89(94.7)**	113(91.1)**	230(92.4)**	459(90.9)**
Antenatal magnesium sulfate	QHH	NA	8(47.1)	69(65.7)	133(73.9)	210(69.5)
	SZH	23(60.5)	78(83.0)*	91(73.4)	174(69.9)	366(72.5)
Cesarean sections	QHH	NA	3(17.6)	44(41.9)	106(58.9)	153(50.7)
	SZH	11(28.9)	36(38.3)	77(62.1)*	166(66.7)	290(57.4)
Facemask ventilation	QHH	NA	4(23.5)	43(41.0)	60(33.3)	107(35.4)
	SZH	2(5.3)	14(14.9)	26(21.0)*	51(20.5)*	93(18.4)**
Tracheal intubation	QHH	NA	12(70.6)	88(83.8)	106(58.9)	206(68.2)
	SZH	36(94.7)	65(69.1)	39(31.5)**	37(14.9)**	177(35.0)**
Chest compressions	QHH	NA	5(29.4)	17(16.2)	24(13.3)	46(15.2)
	SZH	2(5.3)	0(0)**	1(0.8)	0(0)**	3(0.6)**
Epinephrine	QHH	NA	4(23.5)	9(8.6)	9(5.0)	22(7.3)
	SZH	2(5.3)	0(0)**	1(0.8)†	0(0)*	3(0.6)**
PS in DR	QHH	NA	2(11.8)	4(3.8)	3(1.7)	9(3.0)
	SZH	1(2.6)	0(0)†	0(0)	0(0)	1(0.2)†

QHH: Qinghai Red Cross Hospital; SZH: Shenzhen Bao’an Women’s and Children’s Hospital; DR: delivery room; NA: not applicable; PS: pulmonary surfactant; VPI: very preterm infant

Data presented as N (%)

**p < 0.001; *p < 0.01; †*p* < 0.05

### Short-term outcomes

Comparisons of mortality and morbidity between the two cohorts are presented in Table 3. Overall mortality and mortality or major morbidity were significantly higher for 26^0/7^–31^6/7^ WG infants in QHH but not in SZH, and significantly more infants in SZH received active treatment. Among the infants who were actively treated, mortality did not differ between the centers, but mortality or major morbidity was significantly higher in the 28^0/7^–29^6/7^ WG and 30^0/7^–31^6/7^ WG subgroups in SZH. The rates of severe neurological injury and NEC ≥ Stage 2 were not significantly different between the two centers. Moderate or severe BPD was significantly more common in the 28^0/7^–29^6/7^ WG and 30^0/7^–31^6/7^ WG subgroups of QHH infants, but no significant difference between the centers was found in the 26^0/7^–27^6/7^ WG subgroup. The incidence of severe ROP was 66.7% in the 22^0/7^**–**25^6/7^ WG subgroup of SZH infants, and it was significantly higher in the 30^0/7^–31^**6/7**^ WG subgroup of QHH infants. The incidence of LOS was 3.7% in SZH, but no cases of LOS were reported in QHH. After controlling for gestational age and birth weight, an excess risk of adverse outcomes (mortality, mortality or major morbidity, mortality despite active treatment, mortality or major morbidity despite active treatment, moderate or severe BPD, and severe ROP) was observed in the QHH cohort, compared with the SZH cohort.

**Table 3:** Neonatal short-term outcomes

Outcome	Center	22^0/7^ − 25^6/7^ wk	26^0/7^ − 27^6/7^ wk	28^0/7^ − 29^6/7^ wk	30^0/7^ − 31^6/7^ wk	All VPIs	aOR(95%CI)^a^
Mortality	QHH	NA	11/17(64.7)	48/105(45.7)	34/180(18.9)	93/302(30.8)	10.31(6.04–17.61)
	SZH	18/38(47.4)	19/94(20.2)**	11/124(8.9)**	2/249(0.8)**	50/505(9.9)**	1
Mortality or major morbidity	QHH	NA	14/17(82.4)	72/105(68.6)	64/180(35.6)	150/302(49.7)	5.95(4.05–8.74)
	SZH	31/38(81.6)	49/94(52.1) †	20/124(16.1)**	27/249(10.8)**	127/505(25.1)**	1
Active treatment	QHH	NA	9/17(52.9)	60/105(57.1)	141/180(78.3)	210/302(69.5)	-
	SZH	26/38(68.4)	82/94(87.2)*	115/124(92.7)**	245/249(99.2)**	468/505(92.7)**	-
Mortality despite active treatment	QHH	NA	3/9(33.3)	7/60(11.7)	4/141(2.8)	14/210(6.7)	3.14(1.31–7.53)
	SZH	6/26(23.1)	7/82(8.5)	5/115(4.3)	2/245(0.8)	20/468(4.3)	1
Mortality or major morbidity despite active treatment	QHH	NA	6/9(66.7)	30/60(50.0)	34/141(24.1)	70/210(33.3)	3.35(2.17–5.17)
	SZH	19/26(73.1)	37/82(45.1)	14/115(12.2)**	27/245(11.0)*	97/468(20.7)**	1
Severe neurological injury^b^	QHH	NA	2/17(11.8)	3/104(2.9)	7/180(3.9)	12/301(4.0)	1.22(0.53–2.81)
	SZH	9/29(31.0)	8/79(10.1)	3/114(2.6)	5/244(2.0)	25/466(5.4)	1
NEC ≥ Stage 2^c^	QHH	NA	0/12(0)	2/80(2.5)	1/158(0.6)	3/250(1.2)	0.65(0.17–2.52)
	SZH	0/33(0)	4/89(4.5)	1/119(0.8)	5/248(2.0)	10/489(2.0)	1
Moderate or severe BPD^d^	QHH	NA	4/8(50.0)	19/55(34.5)	22/138(15.9)	45/201(22.4)	3.66(2.20–6.06)
	SZH	6/21(28.6)	22/77(28.6)	8/112(7.1)**	15/244(6.1)*	51/454(11.2)**	1
Severe ROP^e^	QHH	NA	2/6(33.3)	4/47(8.5)	4/122(3.3)	10/175(5.7)	3.24(1.19–8.83)
	SZH	14/21(66.7)	15/77(19.5)	3/112(2.7)	0/223(0)†	32/433(7.4)	1
EOS	QHH	NA	0/17(0)	1/105(1.0)	5/180(2.8)	6/302(2.0)	4.25(0.96–18.81)
	SZH	0/38(0)	1/94(1.1)	0/124(0)	2/249(0.8)	3/505(0.6)	1
LOS^f^	QHH	NA	0/12(0)	0/80(0)	0/158(0)	0/250(0)	-
	SZH	2/33(6.1)	6/89(6.7)	4/119(3.4)	6/248(2.4)	18/489(3.7)*	-

QHH: Qinghai Red Cross Hospital; SZH: Shenzhen Bao’an Women’s and Children’s Hospital; VPIs: Very preterm infants; aOR: adjusted odds ratio; NEC: necrotizing enterocolitis; BPD: bronchopulmonary dysplasia; EOS: Early onset sepsis; LOS: late onset sepsis; ROP: retinopathy of prematurity; CI: confidence interval

^a^The results were adjusted for gestational age, multiple pregnancy and antenatal steroid, the reference cohort is the SZH infants

^b^Severe neurological injury incidence was evaluated in infants with neuroimaging results

^c^NEC incidence was evaluated in infants who were hospitalized for more than 3 days

^d^BPD incidence was evaluated in infants who survived with a PMA of 36 weeks

^e^ROP incidence was evaluated in infants who received eye examinations

^f^LOS incidence was evaluated in infants who were hospitalized for more than 3 days

Data are presented as n/N (%) or aOR (95% CI).

**p < 0.001; *p < 0.01; † *p* < 0.05

### Discharge parameters

The discharge parameters, including discharge PMA, weight, length of hospital stay, and total cost of hospitalization are shown in Table 4. No significant differences were found in the discharge PMA, weight, or length of hospital stay between the QHH and SZH infants. However, the total cost of hospitalization was higher in the 28^0/7^-29^6/7^ WG and 30^0/7^-31^6/7^ WG subgroups of QHH infants.

**Table 4:** Discharge parameters of the infants who survived and were discharged after active treatment

Parameters	Center	22^0/7^ − 25^6/7^ wk	26^0/7^ − 27^6/7^ wk	28^0/7^ − 29^6/7^ wk	30^0/7^ − 31^6/7^ wk	All VPIs
No. of infants	QHH	0	6	53	137	196
	SZH	20	75	110	243	448
Discharge PMA (weeks)	QHH	NA	37.50(36.36, 41.79)	38.00(35.64, 39.29)	36.86(35.57, 38.00)	37.14(35.71, 38.68)
	SZH	40.00(38.89, 41.14)	38.57(37.43, 40.00)	37.71(36.29, 38.61)	36.86(35.86, 38.14)	37.43(36.18, 38.71)†
Discharge weight (g)	QHH	NA	2400(2225, 2788)	2320(2170, 2675)^a^	2320(2170, 2490)^b^	2320(2178, 2500)^c^
	SZH	2850(2600, 3230)	2375(2150, 2640)	2315(2115, 2636)	2200(2070, 2400)*	2260(2100, 2560)†
Length of hospital stay (days)	QHH	NA	79(71, 104)	63(45, 73)	40(34, 50)	44(35, 62)
	SZH	104(98, 114)	80(73, 93)	61(52, 69)	41(33, 49)	52(39, 70)**
Total cost (yuan)	QHH	NA	175,969(167,171, 265,668)	147,950(104,232, 170,714)	95,056(69,405, 114,479)	103,577(75,288, 141,405)
	SZH	24,200(211,537, 282,818)	172,209(151,814, 217,344)	117,191(92,305, 137,641)*	66,346(49,543, 89,942)**	94,466(63,245, 139,858)

VPI, very preterm infant; QHH: Qinghai Red Cross Hospital; SZH: Shenzhen Bao’an Women’s and Children’s Hospital; PMA: postmenstrual age; NA, not applicable

^a^discharge weight was available in 49 infants; ^b^discharge weight was available in 131 infants; ^c^discharge weight was available in 186 infants

Data are presented as median (P25, P75)

**p < 0.001; *p < 0.01; †*p* < 0.05

## Discussion

Our study compared care practices for infants born at QHH and SZH between 22^0/7^ and 31^6/7^ WG, as well as their short-term outcomes. The infants admitted to SZH tended to be more immature than their counterparts at QHH, and more infants in SZH were actively treated according to doctor’s advice. More antenatal steroids and cesarean sections were used in SZH, but the intensities of delivery room resuscitations in QHH were higher. Infants born at QHH had higher odds of mortality, mortality or major morbidity, moderate or severe BPD, and severe ROP, even after adjusting for gestational age and birth weight. Among infants who received active treatment at SZH, better outcomes, including mortality and mortality or major morbidity were observed. The total cost of hospitalization for infants 28–31 WG was higher in QHH than in SZH, though their discharge PMA and length of hospital stay were not different between the centers.

More than 25% of infants in the SZH cohort vs. only 5.6% of infants in the QHH cohort were born earlier than 28 WG. The reasons cited for the difference in GA between the cohorts were that fewer extremely preterm infants were born in QHH and a large proportion of them were not resuscitated in the delivery room.

Approximately 30% of the VPIs’ parents in QHH withheld treatment from their infants against medical advice, which was more than 3 times the number of VPIs’ parents that did the same in SZH. The rate of withholding treatment increases as the gestational age decreases. When treatment of these infants in QHH was withdrawn, 70% of them were still under invasive mechanical ventilation, 23% were supported with non-invasive positive pressure ventilation or high flow nasal cannula, while none of these infants was diagnosed with severe neurological injury or NEC. Most of these infants were discharged home in their early days of life, after treatment was withdrawn, and they died without life sustaining support. This is a frustrating situation for healthcare providers; once the parents decide to withhold or withdraw care, we can do nothing but try to persuade them to accept treatment, which seems to be a hopeless effort. This problem is pervasive in China, and the rate of withholding treatment from extremely preterm infants ranges from 49.8 to 16.3%, depending on the socioeconomic level of the local region [11]. The reasons for deciding to withdraw care are complex; for example, a study from southern China revealed that the most important reason was the economic burden and fear of poor or uncertain outcomes [12]. A study from the United States reported that decisions to withdraw treatment from extremely preterm infants were associated with gestational age, race, ethnicity, necrotizing enterocolitis, and severe brain injury [13]. The large gap in economic development between Xining and Shenzhen could have influenced the parents’ concerns about the economic burden of saving their VPIs; however, more studies are needed to investigate parents’ reasons for withholding treatment and to identify possible methods for avoiding unnecessary withholding of treatment from preterm infants.

More than 90% of VPIs were given antenatal steroids in SZH, but less than 75% of VPIs in QHH received them, although we observed an increasing trend in the use of antenatal steroids in QHH as GA increased. The effectiveness of antenatal steroid administration has been confirmed in randomized controlled trials [14], and its use has been recommended in several guidelines [15–17]. Although the rates of antenatal steroid use worldwide continue to increase, differences between countries and regions persist [2, 18, 19]. Given the benefits of antenatal steroid use, measures are needed to increase its use in QHH.

More than half of the VPIs were born via cesarean section in both hospitals, with more cesarean sections performed in SZH. The association between cesarean sections and neonatal outcomes has not been investigated via randomized controlled trials. An observational study conducted in the US of VPIs from 22 to 31 WG, showed significantly reduced odds ratio for neonatal deaths among infants delivered through primary cesarean section between 22 and 25 WG, but no difference was found among infants born later than 26 WG [20]. However, a nationwide cohort study from Korea reported that cesarean section was not associated with any survival or morbidity advantage in very low birth-weight infants [21]. More studies are needed to examine the association between cesarean sections and neonatal outcomes.

More infants born between 28 and 31 WG in QHH received respiratory support from facemask ventilation or tracheal intubation in the delivery room. Evidence from randomized controlled trials shows that avoiding delivery room intubation and stabilizing preterm infants on continuous positive airway pressure reduces deaths and BPD [22, 23]. A quality improvement project decreased the rate of delivery room intubations by increasing the success rate of facemask positive-pressure ventilation; a reduced need for mechanical ventilation and lower rates of BPD and severe ROP were observed after the project [24]. Therefore, QHH could also conduct a similar quality improvement project to reduce their number of delivery room intubations.

The overall mortality and mortality or major morbidity of the infants were higher in the QHH cohort compared to the SZH cohort. This finding can be explained, in part, by the higher proportion of infants subjected to the withdrawing or withholding of treatment in QHH. In order to compare neonatal outcomes (excluding the negative impact of withdrawing or withholding treatment), we analyzed the mortality and mortality or major morbidity of infants who were actively treated. Interestingly, no difference in the mortality of infants after active treatment was found between the two centers, but the rate of mortality or major morbidity remained higher in QHH. Possible explanations for this difference include the high incidence of moderate to severe BPD in QHH and the lower rate of severe ROP in SZH.

We found a much higher rate of moderate or severe BPD in QHH than in SZH. This difference can be attributed to differences in antenatal steroid use and delivery room intubations. Potential differences in invasive and noninvasive respiratory management protocols may have contributed to the higher rate of BPD among the QHH infants. Moreover, the difference between the altitudes of the two centers could be a pivotal factor, as QHH is located on the Qinghai-Tibet Plateau, with an altitude of 2275 m above sea level, whereas SZH is located in southern China, with an altitude of 100 m above sea level. Several observational studies have reported an association of higher altitudes with a higher risk for developing BPD [25, 26], and correction for altitude has been reported to reduce BPD rates significantly [27, 28]. We found that the incidence of severe ROP was significantly higher only in the 30^0/7^–31^6/7^ WG subgroup of the QHH cohort, whereas no difference was found in the other subgroups. This finding can be explained by the lower retinal screening rate and lack of altitude correction for the assessment of BPD in QHH. Therefore, further studies are needed to investigate the incidence of BPD in preterm infants at high altitudes.

Although no difference was found in the discharge PMA or length of hospital stay between the two centers, the total cost of medical care for infants born 28–31 WG was much higher in QHH, which can be explained by the much higher incidence of BPD in QHH; a larger amount of oxygen was used, which could have generated more fees.

To the best of our knowledge, this is the first study to compare care practices for VPIs from two tertiary newborn centers located in the northwest and south of China, and their VPIs’ short-term outcomes. This study found differences in the obstetric and delivery room care practices for VPIs, and in their short-term outcomes. However, this study has several limitations. It was a retrospective study, some data were missing, and some neonatal care practices were not compared. Bias existed since more resources were available at SZH than QHH, including adequately trained staff and neonatologists, and human milk bank. Furthermore, the sample size was small and it was not feasible to calculate the adjusted odds ratios for the GAs of the different subgroups.

## Conclusion

In conclusion, the use of antenatal steroids was low, and tracheal intubations and adverse short-term outcomes were more common in QHH infants than in SZH infants. We found a much higher rate of withdrawing or withholding treatment from all subgroups and a higher cost for infants born at 28–31 WG among the QHH infants. Quality improvement projects are needed to enhance obstetric and delivery room care practices, to decrease oxygen use, and to reduce death rates or morbidity in QHH. More investigations of reasons for withdrawing or withholding treatment are needed, as well as a feasibility study of BPD with altitude correction.

Tables.

## Data Availability

The datasets used and/or analyzed during the current study are not publicly available due to the institution restriction but are available from the corresponding author on reasonable request.

## Abbreviations

BPD: Bronchopulmonary dysplasia.
GA: Gestational age.
IVH: Intraventricular hemorrhage.
LOS: Late-onset sepsis.
NEC: Necrotizing enterocolitis.
PMA: Postmenstrual age.
PPROM: Preterm premature rupture of the membranes.
ROP: Retinopathy of prematurity.
SGA: Small for gestational age.
VPIs: Very preterm infants.
WG: Weeks of gestation.
